# Supplementary material for: Lager Yeast Design Through Meiotic Segregation of a Saccharomyces cerevisiae × Saccharomyces eubayanus Hybrid
Source: Front Fungal Biol. 2021 Sep 16;2:733655. doi: 10.3389/ffunb.2021.733655 (PMC10512403; doi:10.3389/ffunb.2021.733655)
Supplement: Supplementary file 4 [file Data_Sheet_4.PDF]

Supplementary Tables and Figures

Lager yeast design through meiotic segregation of a *Saccharomyces cerevisiae* x *Saccharomyces eubayanus* hybrid

Kristoffer Krogerus[1,2], Frederico Magalhães[1,2], Sandra Castillo[1], Gopal Peddinti[1], Virve Vidgren[1], Matteo De Chiara[3], Jia-Xing Yue[3,$], Gianni Liti[3], Brian Gibson[1,4*]

[1] VTT Technical Research Centre of Finland, Tietotie 2, P.O. Box 1000, FI-02044 VTT, Espoo, Finland

[2]Department of Biotechnology and Chemical Technology, Aalto University, School of Chemical Technology, Kemistintie 1, Aalto, P.O. Box 16100, FI-00076 Espoo, Finland

[3]Institute for Research on Cancer and Ageing of Nice (IRCAN), CNRS UMR 7284, INSERM U1081, University of Nice Sophia Antipolis, Nice, France , 06107 Nice, France

[4]Technische Universität Berlin, Chair of Brewing and Beverage Technology, Ackerstraße 76, 13355 Berlin, Germany

[$]Current Address: State Key Laboratory of Oncology in South China, Collaborative Innovation Center for Cancer Medicine, Sun Yat-sen University Cancer Center, Guangzhou, China.

Keywords:

Lager yeast, *S. eubayanus*, brewing, hybrid, tetraploid, sporulation

*Corresponding author. Tel : +49 (30) 314 27 291, Email: brian.gibson@tu-berlin.de

Supplementary Table S1. Assembly statistics for *S. cerevisiae* A81062

Genome size (bp)	12646885
Contigs	21
Mean (bp)	602232
Median (bp)	586058
N50 (bp)	917785
Largest (bp)	1456411
GC(%)	38.15

Supplementary Table S2. *de novo* SNPs in F1 spore clones of *S. cerevisiae* × *S. eubayanus* A225 hybrid.

Chromosome	Position	Reference allele	Alternative allele	Gene	Amino acid change	A225	A226	A227	A228	A229
Sc_chrI	183704	A	C	YGL053W	Gln24Pro	0/0	0/1	0/0	0	1
Sc_chrI	184911	TAAGA	CAAGT	YAR028W	Met12Leu	0/0	0/0	0/1	0	0
Sc_chrI	218873	G	T	YAL067C	Glu63Asp	0/0	0/0	1/1	1	0
Sc_chrI	218890	G	C	YAL067C	Ser69Thr	0/0	0/0	1/1	1	0
Sc_chrII	791876	AGCA	TGGT	YBR298C	CysSer374ThrThr	0/0	0/0	0/1	0	1
Sc_chrIII	7048	G	C	YAL069W-like	Met57Ile	0	.	.	0	1
Sc_chrIV	1284545	G	A	YDR420W	Val500Ile	0/0	1/1	1/1	0	0
Sc_chrV	584634	T	C	YJL225C-like	Ile291Thr	0/0	0/0	1/1	1	0
Sc_chrVI	42156	C	T	YHR216W	Arg482Lys	0/0	0/1	0/1	0	.
Sc_chrVI	115367	AAGAA	GGGAG	YFL023W	Lys497Arg	0/0	0/0	1/1	1	0
Sc_chrVI	130649	GGGAAAAGGAAAAGGAAAAG	GGGAAAAGGAAAAGGAAAAGGAAAAG	YFL015C	Phe19_Leu20ins-LeuPhe	0/0	0/0	1/1	1	0
Sc_chrVII	844553	G	A	YGR189C	Leu404Phe	0/0	0/0	0/1	0	0
Sc_chrIX	299627	CTCAAATTCAAATT	CTCAAATTCAAATTCAAATTCAAATTCAAATT	YIL031W	Asn408_Ser413dup	0/0	0/1	0/1	0	0
Sc_chrX	8820	C	T	YNL336W	Ala138Val	0/0	0/0	0/1	1	0
Sc_chrXI	677693	CATA	AATG	YBR298C-like	Met90Ile	0/0	0/0	1/1	0	1
Sc_chrXI	677814	A	T	YBR298C-like	Leu50His	0/0	0/0	1/1	0	1
Sc_chrXI	677842	T	G	YBR298C-like	Lys41Gln	0/0	0/0	.	0	1
Sc_chrXII	2376	AGCAGT	GGCACC	YLL064C	Thr17Gly	0/0	0/0	0/1	0	1
Sc_chrXIV	555793	C	A	YNL033W	Leu274Ile	0/0	0/0	0/0	1	1
Sc_chrXIV	692789	CTCCCTAAGT	ATCTCCAAGC	YNR044W	Leu340Pro	0/0	0/0	1/1	1	0
Sc_chrXIV	776965	T	C	YIR042C	Lys76Glu	0/0	0/0	0/0	1	1
Se_chr5	272439	T	G	YER056C	Asn356His	0/0	0/1	0/1	0	0
Se_chr10	14626	A	G	YAL063C-like	Ile933Thr	0/0	0/0	0/1	0	0
Se_chr15	313419	C	G	YOR009W-like	Phe91Leu	0/0	0/1	0/1	0	0

Supplementary Table S3. *de novo* SNPs in G10 single cell isolates derived from the F2 spore clone A235.

Chromosome	Position	Reference allele	Alternative allele	Gene	Amino acid change	A235	A235 G10 1	A235 G10 2	A235 G10 3
Sc_chrV	584552	CT	AC	YJL225C-like	p.Leu264Thr	0/0	0/1	0/0	0/0
Sc_chrV	584565	T	G	YJL225C-like	p.Val268Gly	0/0	0/1	0/0	0/0
Sc_chrVII	386689	TTGAT	TT	YGL062W	p.Asp672del	0/0	0/0	0/0	1/1
Sc_chrX	8832	G	A	YNL336W	p.Arg142Lys	0/0	0/1	0/1	0/1
Sc_chrXII	1050334	CTG	CTGTTG	YLR437C	p.Gln18dup	0/0	.	1/1	0/0

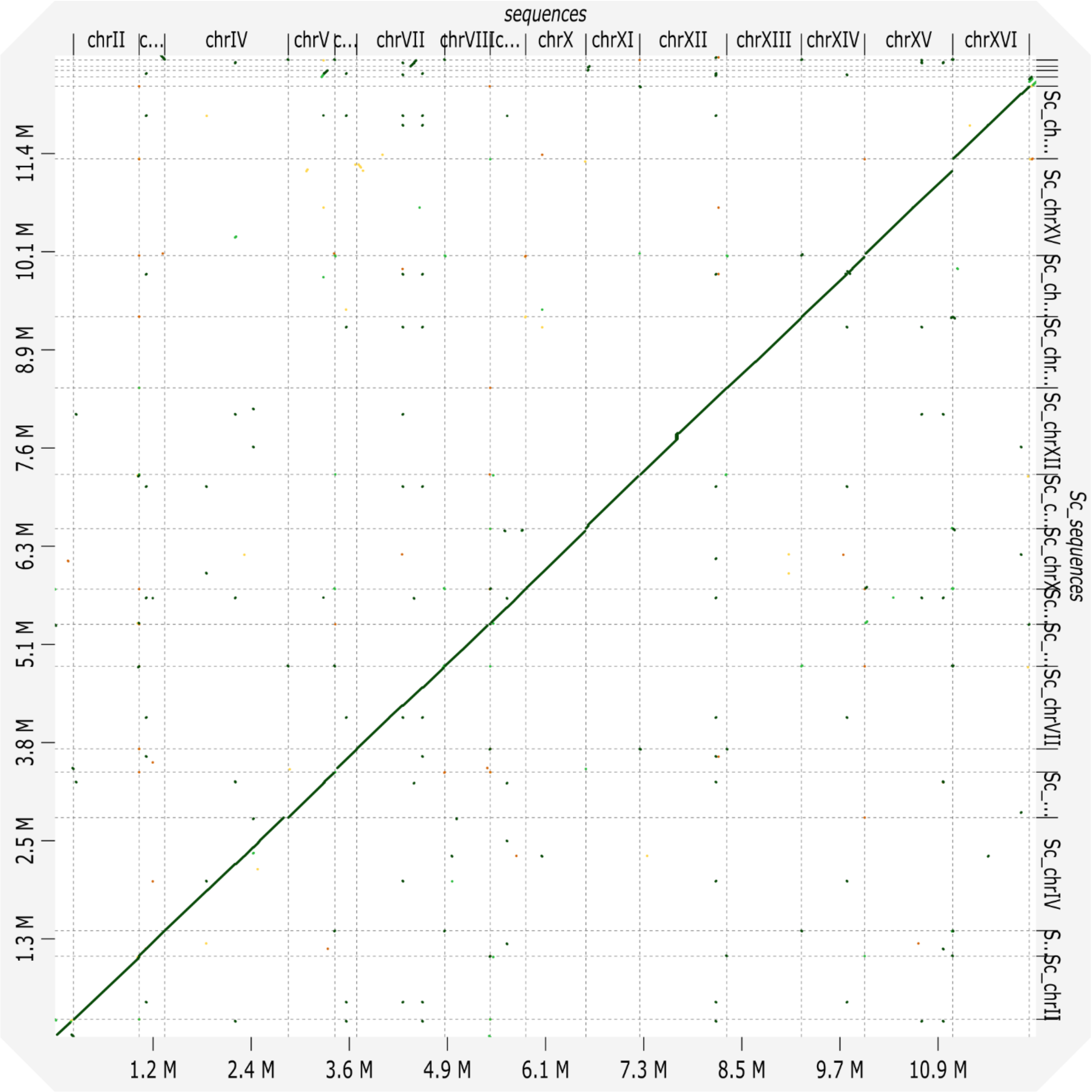

Supplementary Figure S1 - Alignment of *S. cerevisiae* A81062 assembly contigs (y-axis) to *S. cerevisiae* S288C (x-axis)

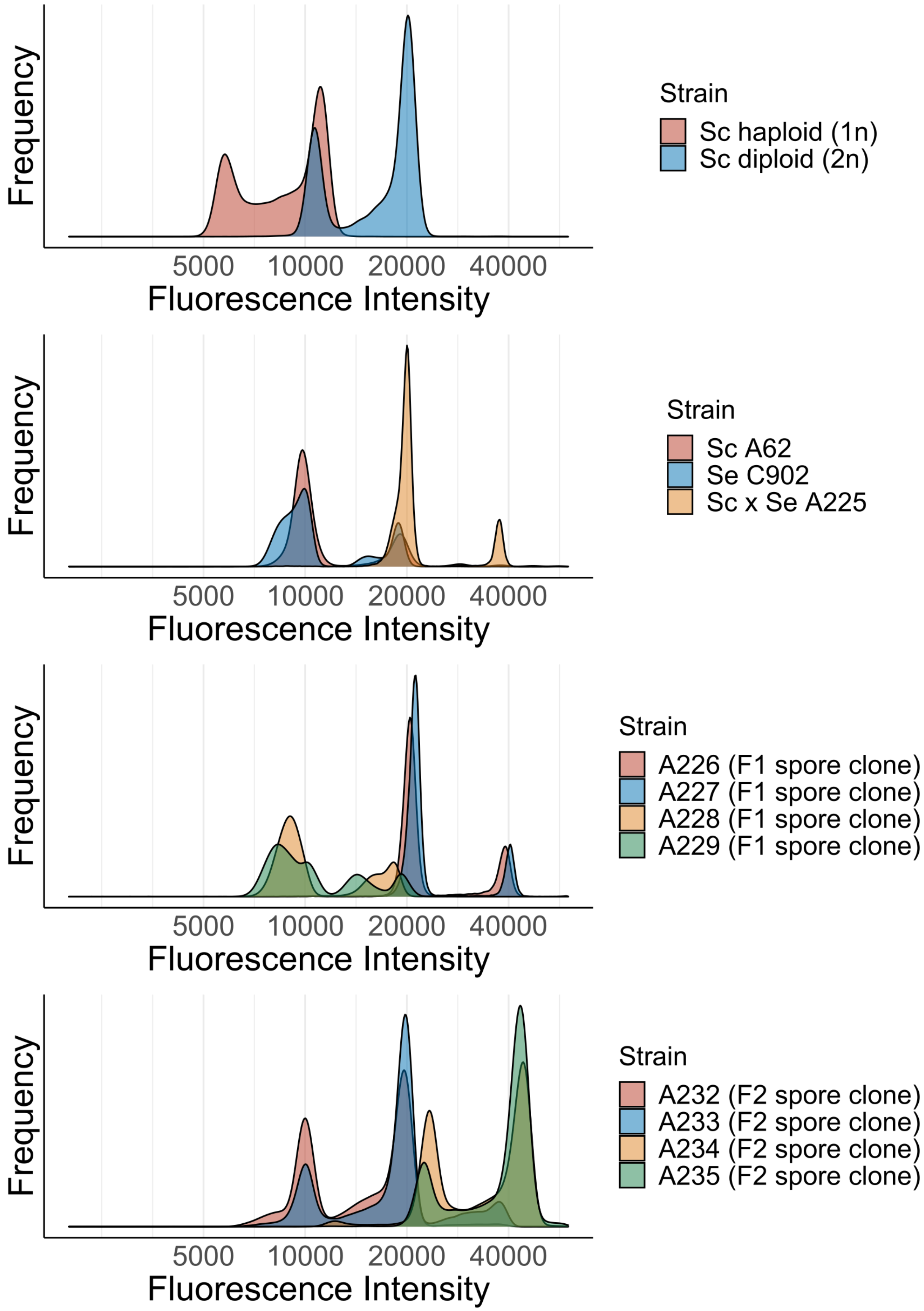

Supplementary Figure S2 - Fluorescence intensity after SYTOX Green-staining

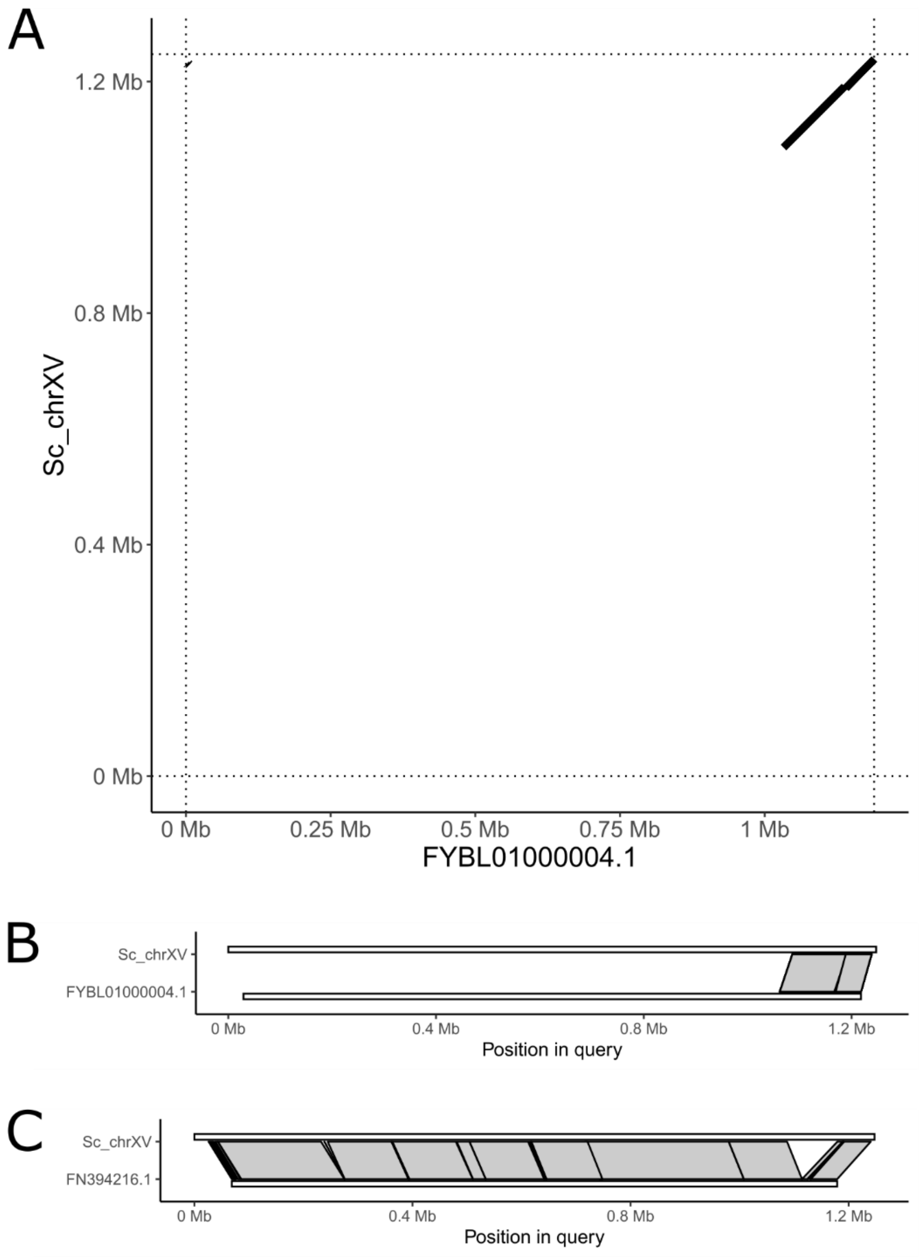

Supplementary Figure S3 - (A and B) Alignment of chrXV of *S. cerevisiae* A81062 to *Torulaspora microellipsoides* scaffold 2 (FYBL01000004.1). (C) Alignment of chrXV of *S. cerevisiae* A81062 to *S. cerevisiae* EC1118 scaffold (FN394216.1)

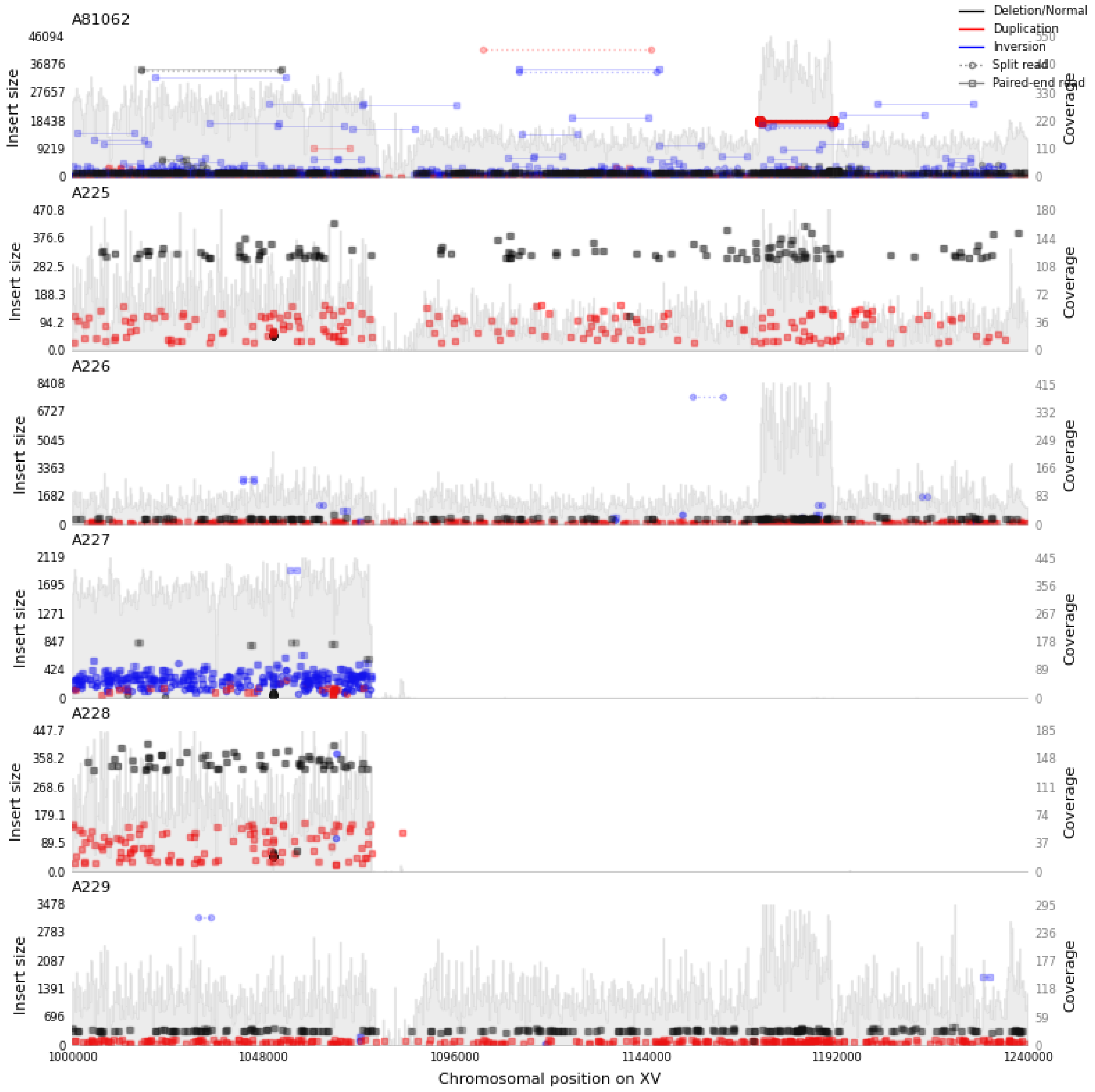

Supplementary Figure S4 - Sequencing coverage on the right arm of *S. cerevisiae* A81062 chrXV in the F1 hybrid A225 and derived F1 spore clones.
